# Dual N-Back Working Memory Training in Healthy Adults: A Randomized Comparison to Processing Speed Training

**DOI:** 10.1371/journal.pone.0151817

**Published:** 2016-04-04

**Authors:** Linette Lawlor-Savage, Vina M. Goghari

**Affiliations:** 1 Department of Psychology, Hotchkiss Brain Institute, University of Calgary, Calgary, Alberta, Canada; 2 Department of Psychiatry, Hotchkiss Brain Institute, University of Calgary, Calgary, Alberta, Canada; University of Akron, UNITED STATES

## Abstract

Enhancing cognitive ability is an attractive concept, particularly for middle-aged adults interested in maintaining cognitive functioning and preventing age-related declines. Computerized working memory training has been investigated as a safe method of cognitive enhancement in younger and older adults, although few studies have considered the potential impact of working memory training on middle-aged adults. This study investigated dual n-back working memory training in healthy adults aged 30–60. Fifty-seven adults completed measures of working memory, processing speed, and fluid intelligence before and after a 5-week web-based dual n-back or active control (processing speed) training program. *Results*: Repeated measures multivariate analysis of variance failed to identify improvements across the three cognitive composites, working memory, processing speed, and fluid intelligence, after training. Follow-up Bayesian analyses supported null findings for training effects for each individual composite. Findings suggest that dual n-back working memory training may not benefit working memory or fluid intelligence in healthy adults. Further investigation is necessary to clarify if other forms of working memory training may be beneficial, and what factors impact training-related benefits, should they occur, in this population.

## Introduction

Enhancing cognitive abilities is attractive for a variety of populations, ranging from individuals experiencing progressive neurocognitive declines (e.g., dementia) through to healthy and accomplished academics [1]. Healthy, middle-aged adults are particularly interested in improving their existing intellectual abilities and potentially staving off age-related cognitive decline [2]. Cognitive enhancement through medication is an intriguing option for healthy adults, although the use of so called “smart drugs” or “academic doping” practices bring forward numerous ethical concerns, including the safety and efficacy of cognitive enhancing medications [1]. However, non-pharmacological options for cognitive enhancement are available, and exponentially growing in popularity [2]. Computerized cognitive training, or “brain-training,” involves structured mental exercises intended to enhance particular cognitive abilities such as attention span and working memory, as well as broader cognitive processes such as general intelligence.

Working memory is a reasonable target of cognitive training given that working memory is a central component of general cognition [3]. Working memory involves temporary storage and processing of information, whether newly perceived, or brought up from longer-term memory stores [4]. The goal of working memory training is to enhance these storage and processing abilities. The dual n-back working memory training task, originally introduced by Jaeggi and colleagues [5], has received notable attention for its potential to improve working memory and other aspects of cognitive performance [5–18]. The dual n-back task requires trainees to respond when visual and/or auditory stimuli match stimuli presented “n” presentations prior. Dual n-back working memory training has been associated with improvement on similar dual n-back tasks, other tasks of working memory, and the broader construct of fluid intelligence [5–18] (the ability to solve novel problems through reasoning and without reliance on previously acquired knowledge [19]).

The theoretical basis for why working memory training might enhance fluid intelligence lies in findings that working memory and fluid intelligence are highly related, yet distinct [3]. Specifically, working memory involves processes such as attending to, holding, and mentally manipulating information (e.g., mentally solving a verbally presented math problem) whereas fluid intelligence invokes higher cognitive abilities such as comprehension, inferential reasoning, and understanding implications of attended to information. Furthermore, working memory and fluid intelligence are each, independently, key aspects of overall intelligence [3], which is consistently associated with academic, social, and vocational success [20,21]. Given the importance of working memory and fluid intelligence to general intelligence, the concept of maintaining or enhancing working memory and fluid intelligence in adults, in a non-pharmacological manner, is enticing and worthy of investigation.

How then, might the dual n-back task increase working memory and broader cognitive abilities? The mismatch model of cognitive plasticity suggests that increasing the demand on cognitive processes leads to expansion of resources associated with cognitive functioning [22]. Based on this model, the ceiling of one’s cognitive abilities can be progressively pushed upward by continually challenging the upper limits of those abilities. Adaptive dual n-back working memory tasks align well with this model. Specifically, as a trainee reaches a pre-specified response accuracy, task difficulty increases. This maintains difficulty at the upper end of the trainee’s ability and, in theory, expands that ability.

Several investigations of healthy young adults support positive effects of dual n-back training. Studies reporting performance gains in tasks such as digit span and reading span [6–8, 10,12] after dual n-back training suggest that training increases ability not only on the trained task (i.e., practice effects), but also on untrained, closely related tasks (near transfer effects). More notable are data suggesting that n-back working memory training transfers to tasks beyond working memory (far transfer effects). Several studies, including a recent meta-analysis, have demonstrated far transfer of dual n-back working memory training to measures of fluid intelligence [14].

However, reported findings are generally inconsistent, and at times, questionable. For example, Redick [23] pointed out that in some cases, statistically significant differences were due not to post-intervention enhancements in the n-back training group, but rather, to post-intervention decreases in control group performance. Furthermore, in several studies comparing dual n-back working memory training to either active or no contact control groups, training related gains were not identified in any one group relative to another for any outcome [24–26]. Other studies demonstrated improvement in near-transfer tasks although no improvement in far transfer [6,8,10,12,15].

Methodological concerns contribute to this inconsistency. For example, sample sizes tend to be small (i.e., often less than 20 per group), and control groups vary among studies (e.g., active versus passive controls, diversity among active control groups). These, and other methodological inconsistencies prompted Au and colleagues [14] to aggregate data from n-back working memory training studies, and investigate the influence of factors such as control groups, training setting (home versus laboratory), geographical location of study (United States versus abroad), and monetary compensation [14]. Analysis of 20 studies (N = 1,022) revealed a small but statistically significant overall Hedges g effect (*g* = 0.24) of n-back training, relative to control conditions, on fluid intelligence outcomes [14]. Effect sizes between active and passive control groups did not significantly differ; however, studies with active control groups demonstrated less differential effect on fluid intelligence than those with passive control groups. Training setting did not influence training effect size; although, studies conducted outside of the United States yielded significantly larger effect sizes than local studies [14]. Finally, amount of financial compensation provided to trainees had a negative effect on training related outcomes [14].

Despite these seemingly positive findings, contentious debate has erupted surrounding this meta-analysis, particularly related to control group type. Dougherty and colleagues [27] conducted a Bayesian analysis of Au and colleagues’ data, which supported the null hypothesis of no training effect relative to active-control groups, although acknowledged training effects when passive controls were utilized. Melby-Lervåg and Hulme [28] argued that Au and colleagues’ meta-analysis failed to account for baseline differences in their effect size calculations, and minimize the difference in effect size between active and passive control. Au and colleagues countered by arguing that type of control group does not influence the effects of n-back training itself, and assert that their initial conclusions stand up to numerous methods of effect size calculation [29]. However, should working memory training groups differ from active control training groups, the benefits of working memory training can be considered more specific to working memory training. Such specificity is important for creating or recommending efficient, targeted training (i.e., treatment) for individuals seeking to improve cognitive performance.

Furthermore, despite Au and colleagues’ [14] attempt to include a wide age range (18–50) in their meta-analysis, healthy adults in dual n-back working memory training studies are consistently of a restricted age range (i.e., mean participant age low to mid 20’s) and often with university affiliation (e.g., psychology undergraduates). This limits the generalizability of findings, whether null or not.

Despite an ever-increasing interest by healthy middle-aged adults to improve their intellectual abilities, or potentially stave off age-related cognitive decline [2], little is known about whether working memory training induces cognitive plasticity in this population. The 30-year to 60-year age range has been comparatively overlooked in cognitive training literature [30]. Enhanced working memory abilities have been demonstrated in middle-aged adults after cognitive, although not specifically working memory, training [30]. Furthermore, Jaeggi and colleagues [31] demonstrated that middle aged adults are less adept at dual n-back tasks than younger adults (aged 19–28), presumably related to the higher memory load required by the dual n-back, relative to single n-back, tasks. This relative deficiency represents an area of potential improvement for middle-aged adults. Also, given that plasticity decreases with age [32], the potential for change in middle-age range adults may be greater than that of older adults. Taken together, there is reason to suggest that middle-aged adults can benefit from dual n-back working memory training.

This study investigates potential cognitive benefits of working memory training in healthy middle-aged adults from a Canadian community. We compare a computerized, home-based dual n-back working memory training program and an active control training program emphasizing processing speed. Processing speed training was chosen as an active comparison because only weak associations have been found between processing speed and either fluid intelligence or working memory [33] and no impact of processing speed training has been found on working memory [34]. Furthermore, a survey of processing speed training studies conducted from 2002–2011 largely indicated that processing speed training transferred to improvements on tasks directly associated with the training task, but did not generally transfer to other tasks [35]. For example, in the ACTIVE study (the data of which contributed to 11 of the 20 surveyed studies), healthy old adults (mean age 73–75) trained for a total of 10 hours across 5–6 weeks on an adaptively speeded visuospatial divided attention task intended to expand participants’ field of view in order to enhance driving abilities. Usual field of view consistently increased after training; however, training effects did not transfer to reasoning, memory, daily problem solving, or other speed based tasks. Processing speed studies that were not part of the ACTIVE study showed similar results [35]. Including this active comparison group rather than a no-training control group allows for all elements of the study with the exception of the specific games used in training (training content) to be controlled for. Last, although no cognitive task is pure, there are processing speed tasks that elicit minimal demand on working memory relative to working memory tasks, which elicit a high working memory load.

Our hypothesis is that relative to the processing speed training control group, the working memory training group will demonstrate post-training improvements in measures of working memory and fluid intelligence.

## Method

### Participants

Healthy adults (*N* = 81) aged 30–60 self-referred to www.braintrainingstudy.ca after learning of the study through postings, a radio interview, and/or social media. Exclusion criteria were history of brain trauma, neurological or psychiatric illness, visual or auditory impairment, benzodiazepine or illicit drug use in the past three months, and pathologies associated with cognitive impairment [36]. Individuals who reported using a dual n-back or processing speed training product in the previous six months, or any Lumosity training program in the previous three months, were also excluded. The University of Calgary Conjoint Faculties Research Ethics Board approved this study, and all participants provided written informed consent.

**Fig 1:**
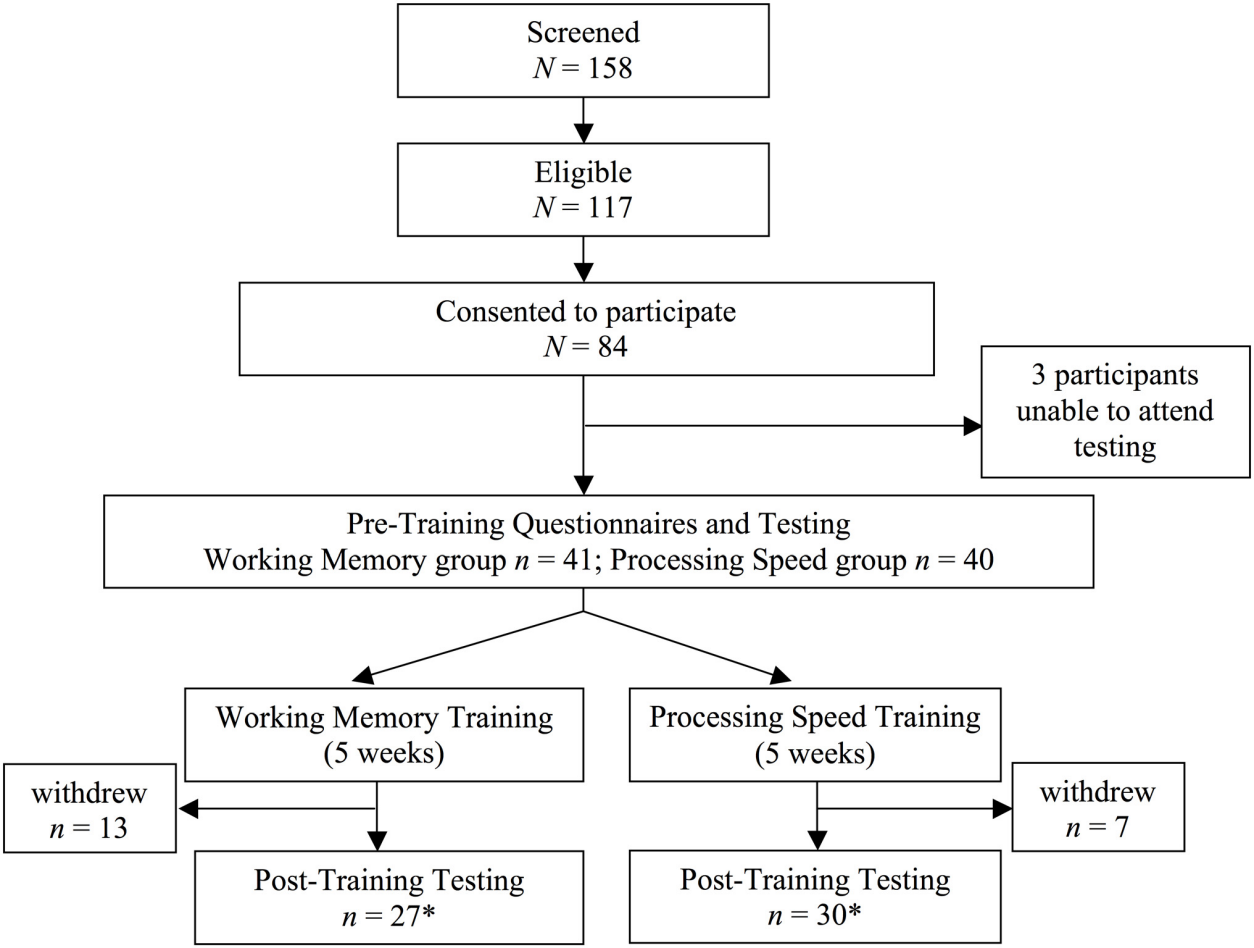
Flow chart of study design. *Data for one working memory training and three processing speed training participants were removed from analysis due to low training dosage, defined as less than 13 of 25 assigned sessions.

Of the 81 healthy adults who consented to participate, 61 (75%) completed baseline and post-training tests. Based on a previous study which identified trends toward far transfer effects after 12 sessions of dual n-back training [5], study participants with less than 12 sessions were excluded from analysis. All remaining participants completed 13 or more training sessions. We also examined the data post-hoc and determined that including participants who completed at least 13 of 25 training sessions did not change whether or not outcomes were significant relative to higher cut-offs (e.g., 14 or more sessions completed). We were therefore able to include more participants in the analysis, without altering gross findings. Therefore, the final analyzed sample consisted of 57 participants (working memory group *n* = 27, processing speed group *n* = 30). See Fig 1 for study design flow chart.

### Training programs

Working memory and processing speed training programs were provided by Lumosity [37] and accessed online. Working memory trainees used the dual n-back, a complex and adaptive working memory training program that simultaneously recruits auditory and visual attention, maintenance, and updating processes. One dual n-back daily training session consisted of 15 rounds of 20+n trials. A trial consisted of a simultaneously presented auditory letter and visual block in one of eight positions in a 3x3 space, lasting 500ms, followed by a 2500ms rest with no stimuli. The trainee responded by button press after each trial (“L” key for visual match and/or “S” key for auditory match), except the first “n” trials. For example, in 3-back, the trainee observed the first three presentations and responded on the fourth. Difficulty level (i.e., “n”) adjusted after each round based on accuracy, such that if accuracy fell below 85%, difficulty would not increase. Participants received feedback after each round to inform them of whether the difficulty would be increasing or remaining the same (e.g., “Nice work. Let’s keep doing 2-back in the next round”). Based on pilot testing, a 15-round training session took approximately 25 minutes to complete, so participants were asked to allow 20–30 minutes to complete each session.

Processing speed training was composed of two simple, visual only, 1-back games requiring participants to quickly determine whether a given symbol (e.g., circle, triangle, square, arrangement of large dots) matched a symbol presented immediately prior. Trainees freely alternated between the two games which differed only in the specific symbols presented. In both games, trainees responded with a left arrow button press for no match, and a right arrow for a match. Symbol presentation was self-paced; once a response was recorded, the next symbol appeared. Participants were instructed to respond quickly. Each game spanned 60 seconds, and number of trials varied by response speed (i.e., faster responders were exposed to more trials within the 60 second game, regardless of accuracy). Due to training platform restrictions, the games were not part of a programmed training session with a prescribed number of games per session; therefore, participants were asked to self-time their daily session, and train for 20–30 minutes per session. Although the speed games recruited immediate visual memory, they did not require maintenance or manipulation (i.e., updating) of information in working memory. Rather, the goal of the game was to respond quickly. Since speed games were based on simple response time and accuracy, stimuli did not increase in difficulty; rather, participants were encouraged to improve their scores (i.e., speed of correct responses) within and across training sessions.

All participants were instructed to complete their 20–30 minute training session in one sitting, 5-days per week, for 5 weeks at a location of their convenience. To encourage adherence, participants completed a log indicating dates and times trained across the 5-week period.

### Measures

#### Baseline measures

At baseline, participants completed an online form assessing demographics (i.e., age, sex, ethnicity, marital status, education, employment, and annual income). Baseline intelligence was estimated during an in-person baseline cognitive testing using the Wechsler Abbreviated Scale of Intelligence—Second Edition (WASI-II; [38]) a short, standardized measure of intelligence yielding a full-scale composite score representing general intelligence. The WASI-II is an update to the original WASI, which has demonstrated convergent and structural validity with more lengthy measures of intelligence [39].

#### Outcome measures

At baseline and post-training, participants underwent cognitive tests measuring working memory, processing speed, and fluid intelligence. Measures were chosen based on their use in previous investigations of working memory training, their ability to reliably measure the cognitive domains of interest, and their sensitivity to age-related differences in cognitive performance.

Auditory working memory was measured with raw scores from the Wechsler Adult Intelligence Scale—Fourth Edition (WAIS-IV) Digit Span subtest [40,41]. Participants repeated verbatim (Digit Span Forward), backward (Digit Span Backward), and in sequential order (Digit Span Sequencing) a series of verbally presented digits. Digit Span Forward is considered a measure of auditory attention span whereas Digit Span Backward and Sequencing recruit the additional working memory component of manipulation (i.e., mentally re-arranging digits).

Working memory was also measured with the Automated Operation Span task [42]. Participants quickly solved mathematical operations presented on screen while remembering, in order, a series of visually presented letters. The derived score was the total number of letters recalled in the correct order. This task is highly correlated with other measures of working memory [33,42]. Furthermore, Automated Operation Span recruits updating processes. For example, an examinee must respond to the math problem, disregard that information while remembering letters, then attend to a new math problem. Both the digit span and operation span tasks require immediate attention, varying degrees of manipulation of information within immediate memory stores, and updating of information. Given that dual n-back tasks also require immediate attention and updating, the additional aspect of manipulation represents a divergent aspect of working memory which can be interpreted as transfer. The working memory tasks were chosen to explore near-transfer effects (i.e., improvements within the same cognitive domain as that which was trained) beyond simply practice effects (i.e., improvement on the same, or very similar, task). Furthermore, including these tasks allowed for investigation of working memory training on attention span, simple working memory (e.g., digit span tasks), and a more complex measure of working memory (i.e., operation span).

Processing speed was measured using raw scores from the Symbol Search and Coding subtests of the WAIS-IV Processing Speed Index [41,42]. Participants quickly responded to visual stimuli by copying symbols on paper and identifying symbols that matched other symbols. Taken together, the Processing Speed Index is a reliable measure of processing speed, and individually, Symbol Search is considered one of the purest measures of processing speed [41].

Two measures of fluid intelligence were utilized in this study: Raven’s Advanced Progressive Matrices (RAPM; [43]) and Cattell’s Culture Fair Test (CCFT) Scale 3 [19]. The RAPM has been used previously to demonstrate working memory training related change in fluid intelligence [11,17,18,44]. For the RAPM, a 6-item practice set progressing from easy to challenging was administered, although not scored, to ensure the participant understood the task. As with previous studies (e.g., [5,10,17,24], the 36 test items were split into parallel odd and even forms counterbalanced across participants with the opposite form delivered at post-test. Participants were allowed 20 minutes to complete the 18-item test. The total number of correct responses formed the dependent variable for this task. For the CCFT, forms A and B were also administered counterbalanced across participants. CCFT Scale 3 contains four subtests: series, classifications, matrices, and conditions providing a measure of fluid intelligence beyond matrices. The CCFT is considered a superior and more specific measure of fluid intelligence compared to measures using only matrix tasks [45,46] and is appropriate for the adults ranging from average to superior intelligence. Raw (total correct) rather than scaled scores were used for analysis given that Cattell & Cattell’s [19] standardized scores and percentiles were based on administration of the forms either sequentially (A followed by B) or in isolation (A or B only) rather than counterbalanced. Our use of raw scores is consistent with other studies that measured CCFT scores over multiple time points (e.g., [47]).

### Procedure

Within five days of completing online consent and questionnaires, participants attended a 2.5-hour baseline cognitive testing session. To reduce carryover and fatigue effects, the administration order of cognitive tasks was counterbalanced [48]. Baseline tests were conducted by the author (LLS) who was blind to randomization until training instructions were revealed at the end of testing. Instructions focused on how to access the online training and the importance of playing only the games described in the instructions (e.g., participants were asked to not independently subscribe to or use other Lumosity games, or other brain training programs). Participants were told only about their training program and were not aware that processing speed and working memory programs were being directly compared, although training program names (e.g., speed match, dual n-back) were not concealed. Game play data were monitored for compliance and reviewed daily to ensure participants were not playing non-assigned games on the Lumosity platform. In rare instances where such contamination was detected, participants were contacted and reminded to only play assigned games, and non-compliant participants were removed from data analysis. Although participants were asked to not engage in other forms of “brain training” during the study period, we were unable to control for such potential contamination. At the conclusion of the training period, participants returned for a 2-hour post-training cognitive testing session composed of the same measures (using parallel forms where indicated) excluding the WASI-II. To maintain experimenter blinding, post-testing was conducted by research assistants trained and supervised by the author (LLS). All testing materials were scripted to ensure consistency of instructions to participants. Participants were asked not to discuss their training program with the research assistants, and assistants did not know specifics of the games or the target skills being trained (e.g., speed versus working memory). Participants were provided with free parking during cognitive testing visits but were not compensated for their time.

### Statistical Analysis

All analyses were conducted using Statistical Package for the Social Sciences (SPSS) version 22, except Bayesian analyses which were conducted using the JASP statistics package, version 0.7, available online at https://jasp-stats.org/ [49]. Correlations (two-tailed) were conducted among all baseline cognitive outcome measures to ensure the chosen measures within each cognitive domain were sufficiently related. Independent samples *t*-tests and chi-squared analyses were used to explore baseline differences in demographics, estimated intelligence, and cognitive outcomes between completers and non-completers, as well as between training groups.

Given moderate correlations between outcome measures within each cognitive domain, cognitive composites were created and analyzed. The working memory composite was created by adding Digit Span and Automated Operation Span z-scores, the processing speed composite from Symbol Search and Coding z-scores, and the fluid intelligence composite from CCFT and RAPM z-scores. Repeated measures multivariate analysis of variance (RM-MANOVA) was used to test for differences in training groups (working memory, processing speed) over time (pre-, post-training) across the three composite dependent variables entered simultaneously.

Post-hoc Bayesian analysis using the Jeffrey-Zellner-Siow (JZS) Bayes factors with default prior scales were conducted to provide more precise probability estimates to support ANOVA results [49–52]. Bayesian analyses compares likelihood estimates of the obtained data occurring under the null versus alternative hypothesis. Advantages and procedures of the Bayesian approach relative to frequentist approaches (e.g., null hypothesis testing using p-values as probability estimates) are extensively discussed elsewhere (e.g., [49–52]). The prior scales utilized to establish Bayes Factors were defaults provided by Rouder and colleagues, and created from independent and multivariate Cauchy distributions [51]. Specific details and formulae can be examined in Rouder and colleagues, who note that resultant default priors are “broadly applicable, computationally convenient, and lead to Bayes factors that have desirable theoretical properties” (pg. 356) [51].

## Results

### Domain Specific Convergent Validity

Two-tailed correlations among cognitive outcome measures at baseline for the full sample (*n* = 81) are presented in Table 1. Statistically significant correlations were revealed for all measures within the working memory domain (*r*’s .33-.80, *p*’s < .001-.02). Similarly, measures within the processing speed domain were significantly correlated, *r* = .67, *p* < .001 and measures within the fluid intelligence domain were significantly correlated, *r* = .30, *p* = .007. WASI-II 4-Item composite scores were significantly correlated with working memory scores at baseline (Digit Span Total, *r* = .35, *p* = .008; Automated Operation Span *r* = .44 *p* = .001, Working Memory Composite scores *r* = .46, *p* < .001).

**Table 1:** Correlations among outcome measures at baseline.

Measure:	Aospan	DSF	DSB	DSS	DST	SS	Coding	RAPM
Aospan	–							
DSF	.33**	–						
DSB	.39***	.48***	–					
DSS	.25**	.35**	.44***	–				
DST	.38**	.78***	.80***	.72***	–			
SS	.02	.06	.14	.20	.21	–		
Coding	.15	-.05	.12	.23*	.14	.67***	–	
RAPM	.13	.21	.13	.13	.19	.20	.16	–
CCFT	.31**	.21	.30**	.22*	.28*	.10	.17	.30**

* p < .05

**p < .01

***p < .001

Aospan = Automated Operation Span; DSF = Digit Span Forward; DSB = Digit Span Backwards; DSS = Digit Span Sequencing; DST = Digit Span Total; SS = Symbol Search; RAPM = Raven’s Advanced Progressive Matrices, CCFT = Cattell’s Culture Fair Test

### Participant Flow

Screening, eligibility, consent, and completion rates for the working memory training and processing speed training groups are presented in Fig 1. Of the 81 participants who began the study, 57 completed all components of the study (pre- and post-testing and at least 13 of 25 training sessions). Of the participants who provided reasons for dropping out or not completing all sessions, primary reasons were being too busy to complete the daily training and unforeseen life events (e.g., unexpected travel, injury, or illness). There were no demographic or cognitive differences between study completers and non-completers.

### Participant Characteristics

Participant characteristics are noted in Table 2. Participants were mostly Caucasian (88%), female (72%), in a coupled relationship (75%), employed full-time (68%), had at least one post-secondary degree or certificate (89%), and incomes above $50,000 per year (74%). All participants spoke and read English fluently, although for 11% of the sample English was not their first language.

**Table 2:** Participant characteristics at baseline.

	Working Memory Group Mean (SD)	Processing Speed Group Mean (SD)	Test (df)	*p*
**N**	27	30	-	-
**Age**	46.63 (8.70)	48.23 (8.96)	*t*(55) = 0.68	.50
**Sex (% female)**	63	80	*χ*^*2*^ (1) = 2.04	.24
**Ethnicity (Caucasian: Hispanic: Asian: Other)**	23: 1: 2: 1	27: 1: 1: 1	*χ*^*2*^ (3) = 0.50	.92
**Marital status (% coupled)**	70	79	*χ*^*2*^ (1) = 0.60	.54
**Education (% post-secondary degree)**	85	93	*χ*^*2*^ (1) = 1.00	.41
**Employment (Full-time: part-time: not employed)**	19: 4: 4	20: 2: 8	*χ*^*2*^ (2) = 1.87	.39
**Income (<$50,000: $50,000-$95,000: >$95,000)**	9: 6: 12	6: 8: 16	*χ*^*2*^ (2) = 1.30	.52
**WASI-II 4-Item Composite**	105.11 (8.07)	104.93 (11.42)	*t*(55) = 0.07	.95

WASI-II = Wechsler Abbreviated Scale of Intelligence (2^nd^ Edition)

At baseline, no significant differences emerged in any demographic variables or cognitive outcome measures between the working memory and processing speed groups. Group means, standard deviations, and confidence intervals for all cognitive measures at baseline and post-training are presented in Table 3.

**Table 3:** Means, standard deviations, and confidence intervals for cognitive outcomes in the working memory (n = 27) and processing speed (n = 29^¶^) training groups.

		Time 1 (Pre-training)		Time 2 (Post-training)	
Task	Group	Mean (SD)	95% CI	Mean (SD)	95% CI
Aospan ^¶^	WM	34.37 (19.73)	26.78, 41.96	42.67 (19.83)	34.74, 50.59
	PS	32.24 (19.62)	24.92, 39.57	38.66 (21.17)	31.01, 46.30
DS	WM	29.74 (4.45)	27.70, 31.78	30.67 (4.42)	28.50, 32.83
	PS	30.13 (5.95)	28.20, 32.07	30.67 (6.50)	28.61, 32.72
SS	WM	35.59 (6.52)	33.13, 38.06	37.78 (6.32)	34.99, 40.57
	PS	34.60 (6.29)	32.26, 36.94	37.30 (7.97)	34.65, 39.95
Coding	WM	75.00 (14.12)	69.95, 80.05	79.93 (15.00)	74.65, 85.20
	PS	72.90 (12.10)	68.11, 77.69	80.40 (12.38)	75.40, 85.40
RAPM	WM	11.37 (2.78)	10.36, 12.38	11.48 (2.98)	10.36, 12.38
	PS	10.56 (2.48)	9.60, 11.52	11.02 (2.34)	10.05, 12.00
CCFT	WM	26.41 (4.38)	24.79, 28.03	29.04 (4.82)	27.23, 30.85
	PS	26.13 (4.02)	24.60, 27.69	28.13 (4.57)	26.42, 29.85

^¶^Data for one processing speed group participant’s post-training scores did not record for the Aospan task, thus processing speed group *n* = 29;

Aospan = Automated Operation Span; DS = Digit Span Total Scores; SS = Symbol Search; RAPM = Raven’s Advanced Progressive Matrices, CCFT = Cattell’s Culture Fair Test; WM = working memory training group, PS = processing speed training group.

### Change in working memory, processing speed, and fluid intelligence

A two-way RM-MANOVA using cognitive composites (working memory, processing speed, fluid intelligence) failed to identify a difference between training groups on cognitive performance over time, *F*(3,52) = 0.37,*p* = .77. A main effect of time was not present, *F*(3,52) = 0.13,*p* = .94.

JZS Bayes factor ANOVAs with default prior scales [49–52] revealed support for the null hypothesis regarding main and interaction effects for all three cognitive outcomes. Specifically, for the working memory composite, the data were 4.80:1 in favour of the alternative hypothesis, or 4.8 times more likely to occur under a model that excluded a main effect of time. Similarly, for the interaction, the data were 33.72:1 in favour of the null. For the processing speed composite, the data were 4.59:1 in favour of the null regarding a main effect of time, and 34.40:1 in favour of the null for the interaction effect. Finally, in the fluid intelligence composite, the data were 4.72:1 in favour of the null for the main effect of time and 39.97:1 in favour of the null for the interaction.

### Training

Working memory trainees spent an average of 17.16 minutes per daily training session, whereas processing speed trainees spent an average of 19.93 minutes per daily training session. Minutes of training were based on actual game-play rather than time spent at the computer; therefore, breaks and time transitioning between games were excluded from training time. Although the two groups did not differ significantly in number of training sessions (working memory group *M* = 22.81, *SD* = 3.27; processing speed group *M* = 22.70, *SD* = 3.35, *t*(55) = 0.13, *p* = .90), a significant difference emerged in total hours of training, *t*(55) = 2.67, *p* < .01 with the processing speed group training more total hours (*M* = 7.54, *SD* = 1.77) than the working memory group (*M* = 6.52, *SD* = 0.93). The two groups did not differ significantly in adhering to the 5-day/week for 5-weeks training schedule based on the number of days which elapsed between the first and last day of training, *t*(55) = -0.33, *p* = .74; working memory training group *M* = 39.37 days, *SD* = 11.19, processing speed training group *M* = 40.33 days, *SD* = 10.80. Utilization of non-assigned games was monitored and contamination was minimal (e.g., 9 of 27 n-back trainees played an average of 6.34 minutes of unassigned games in addition to their n-back training program). Individuals who accessed non-assigned games were contacted and either stopped playing non-assigned games, or were removed from analysis due to low training dosage.

Training progress in the working memory group was based on n-back level achieved. Throughout training, over half the group (52%) reached 4-back, and many reached 5-back (26%). Two participants (7%) did not surpass 3-back and four participants (15%) reached 6-back. Within the working memory group, the mean average n-back level achieved on the first day of training was 1.99 (*SD* = 0.31) and on the last day of training, 3.29 (*SD* = 0.65). The difference in average n-back level achieved from the first to last day of training was statistically significant, *t*(26) = 13.05, *p* < .001.

Training progress in the processing speed group was based on the mean of total scores of the two games on participants’ first and last day of training. The mean of total scores on the first day of training was 4,492.80 (*SD* = 2,626.76) and on the last day of training, 11,773.42 (*SD* = 1,992.28). The difference was statistically significant, *t*(30) = 12.85, *p* < .001.

Total dual n-back game-play time (i.e., training dosage) was positively correlated with improvement on n-back performance across the training period (*r* = .78, *p* < .001). However, larger training dosage did not impact scores on any cognitive outcome measured (*r*’s = -.12-.21, *p*’s = .28-.99). In the processing speed training group, higher training dosage was associated with decreased Automated Operation Span task scores (*r* = -.36, *p* = .04). No other associations were revealed.

Groups did not significantly differ in number of days between last day of training and the post-training cognitive testing, *t*(55) = -1.55, *p* = .13; working memory training group *M* = 13.29 days, *SD* = 15.76; processing speed training group *M* = 7.97 days, *SD* = 9.74. However, these means were skewed by trainees with unusually large lapses between training and post-training tests (working memory trainees with 60 and 62 day lapses, and a processing speed trainee a 47 day lapse). When these outliers were removed from analysis, the groups continued to not significantly differ: working memory training group *M* = 9.48, *SD* = 8.01; processing speed training group *M* = 6.62, *SD* = 6.47, *t*(51) = -1.45, *p* = .15. Due to concern that training effects might have decayed for participants with more, relative to less, days between training and testing, after removing the three outliers, we correlated time lapse with change scores for each cognitive measure. After applying a Bonferroni correction for multiple comparisons, no significant associations were found in either group for any cognitive outcome (working memory training group *r*’s = -.23-.47, *p*’s = .02-.79; processing speed training group *r*’s = -.36-.28, *p*’s = .06-.90). Due to concern that training lapse outliers impacted analysis of the effect of training on cognitive task performance, the RM-MANOVA was conducted with these outliers removed; however, doing so did not impact the results.

## Discussion

This study examined whether dual n-back working memory training, relative to an active control training condition, resulted in improved cognitive abilities, specifically, working memory and fluid intelligence. This investigation was conducted with healthy community adults, aged 30–60, given the increased interest this population has in enhancing cognitive abilities yet lack of empirical examination as to whether working memory training is effective for healthy adults in this age group.

Results indicate that the working memory training group did not improve on transfer outcomes when directly compared to the active control group. Support for the null hypothesis was consistently demonstrated through multivariate analysis of cognitive composite outcomes and Bayesian analyses.

Processing speed training was included in this study as a control condition; therefore, specific hypotheses were not generated. However, based on findings from the ACTIVE study [35], improved processing speed on the trained task (i.e., practice effects) can be expected after processing speed training. In the ACTIVE study, as with ours, processing speed training resulted in improved performance on the trained task although enhancements did not transfer to broader measures of processing speed [35].

Training dosage in the working memory training group, as expected, was positively associated with improved n-back task performance across the 5-week training period. However, no other associations were noted regarding n-back working memory training dose and cognitive outcomes. In the processing speed training group, higher training dosages were associated with poorer Automated Operation Span performance. However, this finding can only be interpreted with caution given that main effects of time were likely not due to training.

Our findings support previous work which fails to replicate near and far transfer benefits of dual n-back working memory training when directly compared to an active control condition (see [24–26]). Although other studies have reported near and far transfer effects, many are called into question due to small sample sizes and problematic control procedures (for thorough discussion see [53–55]). Additionally, a recent reinterpretation of working memory studies that reported positive effects of working memory training relative to control groups revealed that effects were due to decreases in control group performance, rather than reliable increases in training groups [23]. Furthermore, although Au and colleagues [14] concluded positive effects of dual n-back training on working memory and fluid intelligence, a Bayesian approach suggested the data fit better with the null hypothesis [27]. Thus, similar to our data, a Bayesian approach favoured the null.

Our study, and others (e.g., [5,10,17,25]), remain susceptible to criticism based on the measures chosen to detect change. For example, Jaeggi and colleagues [16] conducted an Item Response Theory analysis of the RAPM and concluded that when split into parallel odd and even forms, one form is disproportionately more difficult than the other form, which can distort results by making it appear that one group performed better, or worse, than another group. In this study, we counterbalanced the administration order such that 50% of participants received form A at baseline, and 50% received form B. Although not as precise as an Item Response Theory analysis, we compared mean scores of the two forms at baseline and no significant differences were detected between form A and B, suggesting difficulty of the two forms was similar. However, this does not resolve the issue completely, and future investigators planning to use split forms would be wise to consider examining the difficulty of individual items.

Furthermore, the Automated Operation Span task used in this study may be too divergent from the processes trained by dual n-back. For example, some have suggested that Automated Operation Span relies on recall processes whereas n-back recruits recognition abilities [17]. More recently, Redick and Lindsey [56] suggested that “improving the processes involved in n-back task performance via repeated practice does not result in changes to the processes involved in complex span performance.” This is despite previous data indicating notable correlations between n-back tasks and complex span tasks [57,58].

Lack of training effects in both groups could also relate to motivation or frustration with the training tasks. As noted in the mismatch model of cognitive plasticity, if a training task is too difficult, the trainee may become overwhelmed and give up [22]. Conversely, if a training task is too easy, the trainee may lose interest. It could be that working memory participants found the dual n-back training task too difficult or frustrating to truly push their limits and expand their abilities. Increasing the difficulty of the task immediately after the participant performs well may limit a motivating sense of achievement. In other words, participants’ triumph after a difficult task is rewarded with a more difficult task. Conversely, processing speed trainees may have found the games repetitive and boring, limiting their motivation to improve throughout the training process. However, level of motivation, and reasons for variability in motivation, have not been extensively addressed in dual n-back working memory training studies. To our knowledge, the only investigated factor impacting motivation is monetary compensation [14,16]. Specifically, some findings suggest that paying participants to train decreases intrinsic motivation and results in lower effect sizes [14,16]; although lack of remuneration does not guarantee effects. However, it is possible that rewarding participants for high scores (rather than time spent training) would enhance their persistence and motivation to perform well. Future studies may consider including quantitative or qualitative assessments of perceived training task difficulty throughout the training process, and continual assessment of participant motivation.

As suggested in previous reviews, factors associated with dual n-back training related change are in need of exploration and dissemination [53,54]. Although near and far transfer effects, albeit mixed, have been demonstrated after dual n-back training in younger (e.g., under 30) and older (e.g., over 60) age groups [5–12,15,17,18] effects were not previously investigated in a middle age-range sample. Previous discussions have led to the idea that aging adults may benefit from cognitive training more than younger populations due to loss of complex cognitive performance, including dual n-back performance, with increasing age [31]. Thus, it is reasonable to explore dual n-back as a potential method of increasing complex cognitive abilities. Moreover, given that cognitive plasticity is thought to decline across the aging trajectory [32], middle-age range adults might have a better chance at improving than older adults. However, our null results are more in line with findings that working memory task performance occurs as a monotonic function of age [59].

Other forms of cognitive training have resulted in improved working memory performance for middle-aged adults [30]. Specifically, 23 healthy adults (mean age 53 years) were randomized to a training or no-training control group. The online training program consisted of four exercises targeting visual attention, working memory, and processing speed. Relative to controls, trainees improved on measures of divided visual attention and simple visual memory span. More recently, a large-scale investigation of 4,715 adults ranging in age from 18–80, demonstrated positive effects of a multi-domain training program on both trained and untrained cognitive performance [60]. The authors posit that training with a broad range of tasks increases trainees’ general learning processes and their ability to learn new tasks whether related or unrelated to the trained task. This suggestion is consistent with environmental enrichment theories of learning, and not specific to middle age-range healthy adults [60]. However, training across multiple domains further muddies attempts to identify specific benefits of working memory training. Hence, further research in this field must clearly split into ecologically valid studies meant to identify broad cognitive gains related to some form of training (e.g., investigating and comparing different training programs to determine their effects), or highly controlled laboratory experiments to isolate particular mechanisms of action.

### Limitations

We focused our resources on comparing dual n-back training to an active control group given that numerous previous studies found significant effects of dual n-back training relative to no-contact controls [5–12,15,17,18]. A no-contact control group would allow for comparison of each training program relative to no training, thus controlling for both practice and expectancy effects. In future studies, additional control groups could also provide information about whether some form of training, relative to no-training, is beneficial for this population. In this study, the processing speed training group had a larger training dosage than the working memory group, which may have blunted between-group effects. However, the processing speed training program utilized for this study should not be expected to enhance complex working memory or fluid intelligence. Although we acknowledge that both training programs include working memory and processing speed processes, the extent to which these processes are emphasized within each training program is very different.

It is also possible that with a larger sample size, the differential impacts of the two training programs may be more clearly revealed. Based on a power analysis using G*Power [61], given α = 0.05, power of 0.80 and effect size of 0.24 (as per a meta-analysis of dual n-back training studies [14]) for a repeated measures, within-between interaction, a minimum of 139 individuals would be required for analysis. Alternatively, a lower effect that can be expected when utilizing an active control group is 0.13 [28] yielding a required sample of 467 individuals. Given that similar studies published shortly before our study was planned utilized samples of 20–25 participants per group, we aimed to at least collect a sample with 25 per group (N = 50). Despite the study being underpowered, Lilford & Stevens’ suggest that small studies, provided they are well designed, nevertheless contribute to the topic and field in which they are published [62]. Although this study utilized a larger sample than most, ideal future studies would include a larger sample and additional control conditions in order to resolve these limitations.

### Conclusion

The present study suggests that effects of adaptive dual n-back working memory training do not differ from that of active (placebo) control training in healthy middle-aged adults in a self-directed training context. Methodological decisions (e.g., difficulty of individual items across parallel forms, time spent training, measures of frustration or perceived training task difficulty, remuneration for performance, and inclusion of the Bayesian approaches to hypothesis testing) may enlighten future studies. Additionally, future studies could investigate whether single-domain training programs are sufficient or if multiple-domain training is necessary for cognitive improvements to occur in healthy adults.

This study is important as it is the first to explore transfer of dual n-back working memory training specifically in middle age-ranged adults, a population particularly interested in building up cognitive reserve. Perhaps we set ourselves up for null findings by investigating a healthy sample near the peak of their intellectual ability, and a control group that was similar in all aspects except cognitive load. Had support been for the alternative hypothesis, the robustness of dual n-back working memory training given our experimental conditions would have been extraordinary. In this case, support for the null under these experimental conditions is strong. Previous literature has both supported and refuted the effects of targeted working memory training. Working memory training may be safer than pharmacology; however, the impact of working memory training as a method of enhancing cognitive performance remains questionable.
